# Investigating the Impact of Maternal Stress on Milk Glucocorticoids: A Multimethod Approach

**DOI:** 10.1111/psyp.70150

**Published:** 2025-09-16

**Authors:** H. Lustermans, R. Beijers, C. de Weerth

**Affiliations:** ^1^ Radboud University Medical Center, Donders Institute for Brain, Cognition and Behaviour Department of Cognitive Neuroscience Nijmegen the Netherlands; ^2^ Department of Social Development, Behavioural Science Institute Radboud University Nijmegen the Netherlands

**Keywords:** breastfeeding, glucocorticoids, human milk, lactocrine programming, maternal stress

## Abstract

Milk glucocorticoids (MGCs) in human milk may play a pivotal role in the health and development of children. Though MGCs might be increased by maternal stress, prior research yielded conflicting findings. The aim of this preregistered study is to examine the impact of maternal stress on MGCs. Mothers from the low‐risk SMILEY cohort participated 6‐8 weeks postpartum. During a naturalistic study, mothers (*n* = 110) reported their current global affective state three times a day (morning *n* = 96, afternoon *n* = 98, evening *n* = 97) and simultaneously collected milk samples (morning *n* = 89, afternoon *n* = 93, evening *n* = 88). During an experimental study in the lab, mothers (*n* = 80) were exposed to the Trier Social Stress Test or a control task and collected a milk sample 15 min thereafter. Global affect reactivity was measured using visual analogue scales and cortisol reactivity with salivary sampling. Mothers reported on mental health symptoms during the last week(s) using questionnaires. Outcomes were milk cortisol and cortisone, and the cortisol‐to‐cortisone ratio (cc‐ratio). In the naturalistic study, global affect was unrelated to MGCs and cc‐ratio. In the experimental study, the stress condition significantly increased MGCs and cc‐ratio, compared to the control condition. Moreover, irrespective of group, heightened salivary cortisol reactivity was associated with increased MGCs and cc‐ratio, while heightened global affect reactivity was related to increased milk cortisone only in mothers with fewer mental health symptoms. These findings show a causal effect of maternal stressor exposure on MGCs, though replication studies are warranted. Links between MGCs and self‐reported global affect remain unclear, requiring future research.

## IMPACT STATEMENT

Although previous studies have reported mixed results regarding the link between maternal stress and human milk glucocorticoids, our findings are the first to demonstrate a causal effect of acute stressor exposure on milk glucocorticoids, along with salivary cortisol reactivity. In contrast, we found limited evidence connecting global affect (reactivity) to milk glucocorticoids in both naturalistic and experimental settings.

## 1 Introduction

Breastfeeding, and the various nutrients in human milk, have undeniable long‐term health benefits for infants (Hernández‐Luengo et al. 2022; McGowan and Bland 2023; Pérez‐Escamilla et al. 2023; Victora et al. 2016). Human milk not only contains nutrients, such as fats, proteins and micronutrients; milk also consists of non‐nutritive components, such as immune factors, microbes and hormones (de Weerth et al. 2022). Growing evidence suggests that non‐nutrient bioactive factors contribute to the long‐term development of child behavior and cognition, a process termed Lactocrine Programming (Bartol et al. 2008; Hinde et al. 2013). The major glucocorticoids in milk, cortisol and cortisone, are recognized as potential relevant contributors to this process and vary greatly in concentration from one mother to another (de Weerth et al. 2022). Hence, milk glucocorticoids (MGCs) could act as a mechanism for transmitting important information from the mother to the infant, as neonates are adapted to receive and use maternal milk hormonal signals for development (de Weerth et al. 2022). In this context, note that formula does not contain any MGCs. Given that milk glucocorticoids likely originate from maternal systemic circulation (Kervezee et al. 2024), maternal factors may influence concentrations of milk cortisol and its inactive metabolite, cortisone. One such factor that might influence MGC concentrations might be maternal stress, although results from prior research regarding the association between maternal stress and MGCs are mixed (Aparicio et al. 2020; Lindberg et al. 2021; Romijn et al. 2021; Tekgündüz et al. 2023; Zielinska‐Pukos et al. 2022). One reason for these inconsistent findings might be that these results stem mostly from observational studies. Therefore, the aim of this study is to examine the associations of maternal stress with MGCs within a naturalistic and an experimental study.

Glucocorticoids are essential cholesterol‐derived signaling hormones that orchestrate neurobiological and cognitive functions and that play a fundamental role in the maintenance of homeostasis by regulating physiological processes such as metabolism, immune response, and reproduction (Timmermans et al. 2019). Animal studies indicate that glucocorticoids are also involved in the development of the mammary gland and are important for milk synthesis and secretory differentiation and activation (Hannan et al. 2023; Stead et al. 2022). In humans, glucocorticoids are produced in the adrenal glands as the output of the hypothalamic–pituitary–adrenal (HPA) axis and follow a circadian rhythm (Spencer and Deak 2017). Free cortisol in mother's systematic circulation likely diffuses passively into her milk. Subsequently, the mammary gland synthesizes the inactive metabolite cortisone out of cortisol by the action of the enzyme 11β‐hydroxysteroid dehydrogenase 2 (11β‐HSD2; Hollanders et al. 2017). Milk cortisol and cortisone show a similar circadian rhythm as in maternal circulation, including a morning peak and a nadir in the evening (Pundir et al. 2017; van der Voorn et al. 2016). Importantly, during the first postpartum months, human infants have not yet fully developed a circadian rhythm in their own production of glucocorticoids (Kervezee et al. 2024). Hence, the MGCs reflect maternal systemic glucocorticoids' circadian rhythm and might contribute to infant circadian rhythm development.

As the activity of the HPA axis and its release of cortisol is further increased upon physiological and psychological stressors (Timmermans et al. 2019), maternal stress is expected to be a determinant of MGCs (de Weerth et al. 2022; Stead et al. 2022). Indeed, maternal self‐reported symptoms of stress on postpartum day seven were associated with increased milk cortisol sampled on the same day (Tekgündüz et al. 2023), and self‐reported psychological distress at 6 weeks postpartum was associated with higher milk cortisol in the first three postpartum months (Aparicio et al. 2020). In line with this, relaxation therapy lowered milk cortisol concentrations at two weeks postpartum (Mohd Shukri et al. 2019). However, not all studies find evidence for a link between maternal stress and MGCs. For example, no association was found between milk cortisol at two months postpartum and self‐reported stress at three months postpartum (Lindberg et al. 2021) or between milk cortisol and self‐reported stress assessed at 1, 3, and 6 months postpartum (Zielinska‐Pukos et al. 2022). Furthermore, Romijn et al. (2021) found that a group of women with high symptoms of anxiety and depression had significantly lower milk cortisol over a single day at 1 month postpartum, compared to a control group without these symptoms (Romijn et al. 2021). The inconsistencies in results might be due to the large heterogeneity in study designs. In particular, the time interval between the assessment of maternal stress and collection of milk sample(s) was highly variable (ranging from a few hours to several weeks). Also, the frequency and timing of milk sampling throughout the day varied greatly. Lastly, prior research mostly studied milk cortisol, while in human milk there is a higher abundance of cortisone. There is very limited knowledge on how maternal stress is related to cortisone levels, or the cortisol‐to‐cortisone ratio (CC‐ratio, reflecting the activity of 11β‐HSD2) and its significance for offspring development.

To obtain more insight into the impact of maternal stress and MGCs (i.e., cortisol, cortisone) and the CC‐ratio, we carried out two studies within the same cohort at around 6 to 8 weeks postpartum. Main hypotheses and analyses were preregistered on the Open Science Framework (osf.io/ndw8j). First, a naturalistic home study was carried out in which mothers reported their current global affective state three times in 1 day and simultaneously collected milk samples. We hypothesized that a higher global affect score would be associated with higher concurrent concentrations of MGCs. Second, an experimental study (randomized controlled trial; RCT) was carried out to test the causal effect of a laboratory acute psychosocial stressor on post‐stressor MGCs. We hypothesized that post‐stressor MGCs would be significantly higher in the laboratory stressor group compared to the control group. Moreover, based on the fact that frequent or prolonged exposure to psychosocial stress can dysregulate the HPA axis (Herman et al. 2016), we investigated whether mental health symptoms (i.e., anxiety, depression, stress) during the last week(s) would moderate the effects of the laboratory stressor on post‐stressor MGCs. Prolonged exposure to stress is thought to increase the sensitivity of the adrenal cortex to ACTH, amplifying glucocorticoid response to certain stressors (Herman et al. 2016). Therefore, we hypothesized that women who experience high levels of mental health symptoms would have the strongest increase in post‐stressor MGCs (i.e., hyperreactivity of the HPA axis) in response to the laboratory stressor. We included general as well as postpartum‐specific mental health symptoms since challenges in the postpartum period are not always captured by questionnaires assessing general mental health symptoms. Lastly, within the experimental study, mothers were separated from their young infant, which might have been stressful by itself. Therefore, we also investigated whether maternal stress reactivity (i.e., global affect and salivary cortisol) during the laboratory visit was associated with post‐stressor MGCs, irrespective of which experimental group the mother was assigned to. We hypothesized that higher stress reactivity during the laboratory visit would be associated with higher post‐stressor MGCs and that this association would be strongest for mothers experiencing high levels of mental health symptoms.

## 2 Method

### 2.1 Participants

**FIGURE 1:**
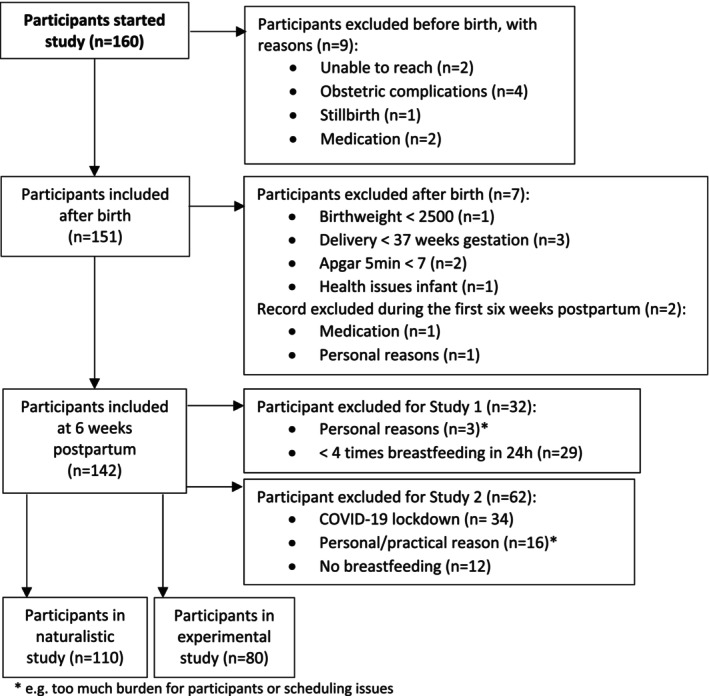
Flowchart of study participants.

Participants are mothers from the SMILEY cohort (Study of Microbiota and Lifestyle in the Early Years): a healthy community sample of Dutch mother‐infant dyads (*N* = 160) followed from mid‐pregnancy to 2 years of age. At enrollment, inclusion criteria for the pregnant women were: ≥ 18 years of age, mastery of the Dutch language, singleton pregnancy, and pre‐pregnancy BMI ≤ 30 kg/m^2^. During the study, mother‐infant dyads were excluded in case of severe obstetric complications, severe maternal mental or physical health issues, preterm delivery (i.e., < 37 weeks of gestation or < 2500 g; WHO), and severe developmental abnormalities. An additional inclusion criterion for the naturalistic study was mothers breastfeeding directly from the breast for at least four feedings a day (24 h). For the experimental study, the inclusion criterion was mothers giving any breastfeeding to their infant. At 6 weeks postpartum, a subgroup of 110 mothers collected data for the naturalistic study, and at 8 weeks postpartum, a subgroup of 80 mothers participated in the experimental lab study (for a flowchart of the sample sizes, see Figure 1). Post hoc power analyses showed that for Study 1, medium effect sizes could be detected with a power of 0.93 (considering a linear regression model and alpha of 0.05) and for Study 2, that medium effect sizes could be detected with a power ranging from 0.73 to 0.82 (considering linear regression analyses with three to four predictors and alpha of 0.05). Prior to data collection, mothers gave written informed consent.

### 2.2 Naturalistic Study

#### 2.2.1 Procedure

At 6 weeks postpartum (*M* = 6.51, SD = 0.47), mothers were asked to report on their current global affective state and collect concurrent milk samples during 1 day. This was done three times within given timeslots: 7:00 AM–10:00 AM (i.e., morning), between 11:00 AM and 2:00 PM (i.e., afternoon) and between 7:00 PM and 11:00 PM (i.e., evening). Moreover, the maximum accepted time between the global affect assessment and milk sample was 45 min.

#### 2.2.2 Measures

##### 2.2.2.1 Global Affect

To assess short‐term fluctuations in global affect (e.g., within a day or during a laboratory manipulation), mothers completed the Global Affect scale by Monk (1989), a paper‐based visual analogue scale (VAS) with four unipolar items measuring sadness, tenseness, happiness, and calmness. This scale was chosen because it ensures consistency (e.g., calmness and tenseness should correlate negatively) and is quick to complete (under a minute), reducing participant burden and minimizing bias caused by fatigue. The visual analogue format also enhances data accuracy by avoiding forced‐choice responses and reducing the influence of previous answers. For each of the four feelings, participants were asked to place a cross on a line of around 10 cm to indicate how they felt at that moment, ranging from “not at all” at the outer left to “a lot” at the outer right. Scores were calculated by measuring the distance in millimeters from the start of the line to the cross and recoded to standardized ranges of 0–100. The global affect score was calculated by the following formula: [(sad) + (tense) + 200 – (happy) – (calm)]/4. A higher score represented more self‐reported global affect (range 0–100).

##### 2.2.2.2 Milk Glucocorticoids

To assess milk glucocorticoids, mothers were asked to collect a ~4 mL sample of milk. Mothers were instructed to collect the sample prior to breastfeeding their infant within the given timeslot, to ensure all samples were foremilk. Because of other research purposes, the morning sample had to be collected by hand expression. For the afternoon and evening samples, mothers were allowed to choose a method of milk collection themselves (e.g., using a sterilized pump or hand expression). Milk samples were kept in a freezer at home and brought in a cooler to the lab visit, where they were stored in a freezer at −20°C. Subsequently, all samples were brought by a researcher in temperature‐controlled boxes to Utrecht University Medical Centre to determine cortisol and cortisone concentrations, using LC–MS/MS. The CC‐ratio (i.e., cortisol‐to‐cortisone ratio) was calculated by dividing cortisol by cortisone, with a higher ratio meaning more cortisol compared to cortisone.

##### 2.2.2.3 Confounders

The following confounders were included in the analyses: infant age in weeks and the time of sampling.

#### 2.2.3 Missing Data

Some variables contained missing data, namely global affect morning (*n =* 3), global affect evening (*n* = 2), milk sample morning (*n =* 10), milk sample afternoon (*n =* 4) and milk sample evening (*n* = 13). Furthermore, as preregistered, data was thoroughly screened for deviations from the instructed data collection procedure. If participants did not collect the global affect measure and the milk sample within 45 min, the available global affect and milk cortisol/cortisone data was recoded into missing data (morning *n* = 12, afternoon *n* = 11, evening *n* = 10). Finally, one participant's afternoon and evening data was considered invalid due to the use of a prescription cream on the breast prior to milk sampling, and another participant's afternoon and evening data was considered invalid due to unreliable reporting of sampling times. These data have been recoded into missing data. Next, data was inspected for implausible milk cortisol and cortisone values (Miller et al. 2013), which led to one participant's milk cortisol and cortisone data from the whole day being recoded into missing data. Lastly, the remaining data was inspected for outliers (i.e., values of more than 3 standard deviations above/below the mean), which were considered valid data points (global affect morning *n =* 1, global affect afternoon *n =* 2, milk cortisol *n* = 4, milk cortisone *n* = 1). Regarding confounders, two outliers (infant age (*n* = 1) and time of sampling (*n* = 1)) were recoded into missing data. The missing data was imputed by means of multiple imputation using the mice package (van Buuren and Groothuis‐Oudshoorn 2011), which is considered an effective technique to minimize the bias of missing data on parameter estimates (Enders 2017; Woods et al. 2024). Final results are the pooled parameter estimates based on five imputed datasets (van Buuren 2018). As a sensitivity analysis, these results were compared with results from analyses using list‐wise deletion (i.e., including only complete cases).

#### 2.2.4 Statistical Analyses

All analyses were performed in R. The distribution of the milk cortisol data (i.e., morning, afternoon, evening) was positively skewed and therefore these data were log transformed. To test the preregistered hypotheses, we ran a linear regression model for each timepoint (i.e., morning, afternoon, evening), with milk cortisol as the outcome and global affect, infant age, and time of sampling as predictors. These models were repeated with milk cortisone as an outcome. Exploratively, we ran the models a third time with the CC‐ratio as an outcome. The models were inspected for homogeneity and distribution of the residuals. Outliers of model residuals were visually inspected and their influence was tested using Cook's Distance.

Note that at the stage of preregistration, a multilevel approach was first considered. However, linear regression analyses were considered the best approach. The reasons for this were the lack of clustering of the data within participants (i.e., multilevel modeling is used to deal with dependence of observations, see Ntani et al. 2021), the maximum of three observations for each participant (i.e., five observations are recommended as minimum; see Asampana Asosega et al. 2024), and the fact that our hypotheses were targeted at links between maternal stress and milk glucocorticoids within specific moments in time. Nonetheless, as a sensitivity analysis, we performed multilevel models. The timepoints during the day (i.e., morning, afternoon, evening) were introduced at Level 1 and nested within the mothers at Level 2. Subsequently, the multilevel models were built by adding variables one by one. After adding a new variable, improvements to the model were examined using maximum log‐likelihood. If a variable did not significantly improve the model, it was excluded from the model. First, the fixed and random effects of linear time were separately entered into the model. Quadratic time effects were not considered, as the three timepoints did not allow for their estimation. Thereafter, infant age was added to the model as a fixed effect. Lastly, the predictor global affect was added as a fixed effect. The results of the final models led to similar results as the linear regression analyses (Table S4).

### 2.3 Experimental Study

#### 2.3.1 Procedure

**FIGURE 2:**
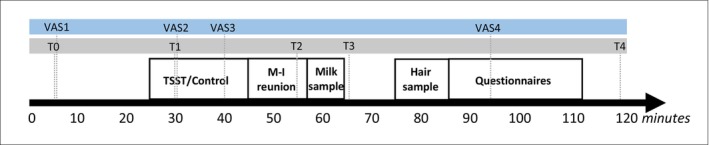
Timeline laboratory visit. M‐I reunion, mother‐infant reunion; T0–T4, Saliva samples; TSST, Trier Social Stress Test; VAS1–VAS4, Visual Analogue Scales. This timeline reflects the study protocol without interruptions (i.e., due to infant feeding or diaper change), however, adaptations were made to meet the infant's needs (e.g., infant feeding prior to manipulation or after sampling milk). Preregistered rules determined if deviations of the protocol were acceptable (e.g., milk samples should be collected ±10 min of the planned time). Note that the hair sample and questionnaires are not part of the current study. Adapted from Bruinhof et al. (submitted).

At 6 weeks postpartum (*M* = 6.22, SD = 0.39), mothers completed online surveys on general and postpartum‐specific mental health symptoms. Subsequently, at 8 weeks postpartum (*M* = 8.20, SD = 1.26), mothers and their infants were invited to the lab; see Figure 2 for a timeline. After an introduction, mothers were shortly separated from their infants for an experimental manipulation. Mothers randomly underwent either an adapted version of a laboratory stress test to induce acute psychosocial stress or a control task. Meanwhile, a babysitter (i.e., a research assistant) or a person accompanying the mother (i.e., partner, friend, family), took care of the infant. After the manipulation, mothers were reunited with their infants. At 15 min after the end of the experimental manipulation, mothers collected a milk sample. Throughout the visit, participants completed four visual analogue scales to assess global affect and collected four saliva samples to measure salivary cortisol concentrations. At the start of the visit, the researcher explained that the protocol of the lab visit was flexible to interruptions to meet the infant's needs, such as a diaper change or feeding. Mothers were informed about the preferable moments for interruptions, namely prior to the experimental manipulation or after the collection of the milk sample. The lab visit lasted 2 h. Because of the cortisol circadian rhythm with its associated high cortisol concentrations in the morning, the lab visit was scheduled in the afternoon (between 12:00 h and 17:30 h) (de Weerth and Buitelaar 2005; van der Voorn et al. 2016).

#### 2.3.2 Randomization

During pregnancy, participants were randomized into two groups: the stressor group and the control group (Lustermans et al. 2024). A stratified random block randomization method with a 1:1 allocation was used to ensure a balance between the stressor and control conditions for the primiparous versus multiparous mothers. The block sizes were randomly chosen from the set of 4 and 6. An independent researcher, who was not involved in the SMILEY study, prepared and concealed the allocation sequence in stapled envelopes. Prior to a lab visit during pregnancy, the researcher opened the envelope and assigned the participant to the stressor group or the control group. Participants that were randomized in the stressor group for the prenatal lab visit were placed in the control group for the postpartum lab visit and vice versa. Participants were blinded to group allocation and were not aware of different group treatments. Debriefing took place at a measurement session at twelve weeks postpartum.

#### 2.3.3 Experimental Manipulation

Stressor: Mothers in the stressor group participated in the Trier Social Stress Test (TSST, Kirschbaum et al. 1993). This laboratory stress test has been shown to be efficient in producing a stress response in most participants (Gunnar et al. 2021). Participants were instructed to hold a five‐minute‐long presentation in which they introduced themselves as a candidate for their dream job in front of a jury. After 5 min of preparation time on their own, participants were led to a room with a large screen on the wall where the jury was present in real time via an online meeting. The two (female) judges, wearing white lab coats, did not give verbal or facial feedback. After the presentation, the jury provided instructions for a mental arithmetic task, which also lasted for 5 min. Participants were not informed about the duration of this part of the task. The performance was recorded by a visible video camera. After the presentation and arithmetic task, participants were instructed by the researcher to wait alone while the jury discussed their performance in private. This waiting lasted for 5 min. The TSST was completed when the researcher came back and told the participant the jury had positively evaluated her performance. Afterwards, mothers were reunited with their infants. The TSST was slightly modified for the current study as follows: (1) because mothers might experience physical complaints related to having given birth ±8 weeks earlier, participants were seated instead of standing, and (2) because of COVID‐19 regulations, the jury was online. An online version of the TSST has been proven to be effective in inducing stress (Gunnar et al. 2021; Meier et al. 2022).

Control: The participants in the control group, while being seated, completed a paper questionnaire about different pieces of fabrics (e.g., denim, lace) that were provided during the task to touch and inspect (van Strien et al. 2014). The task took place in the same room as the TSST. However, participants were not observed by a jury and there was no video camera in the room. Participants were told that the task would last 10 min, but that they did not need to keep an eye on the time, since the researcher would come back when the task was over. Similar to the stressor group, participants had 5 min to prepare for this task by reading the instructions and were asked to wait for 5 min after the task, before reunion with their infant.

#### 2.3.4 Measures

##### 2.3.4.1 Stress Reactivity During the Lab Visit

###### 2.3.4.1.1 Global Affect

To measure global affect during the lab visit, the Global Affect scale by Monk (1989) was used (described in section 2.2.1). These visual analogue scales were completed four times during the lab visit, and the higher the score, the more global affect was reported (range 0–100). VAS1 measured baseline global affect, VAS2 and VAS3 measured global affect during the experimental manipulation, and VAS4 measured global affect during recovery.

###### 2.3.4.1.2 Salivary Cortisol

Five saliva samples were collected to measure cortisol concentrations (see Figure 2 for the precise times). For each sample, participants were asked to carefully collect saliva in a tube using a small straw. Since a cortisol response reaches its peak in saliva 20–30 min post‐stressor (Dickerson and Kemeny 2004; Lopez‐Duran et al. 2014), each sample was taken 25 min later than the moment of interest. Hence, the samples taken at T0 and T1 reflected baseline salivary cortisol concentrations, samples T2 and T3 reflected the experimental manipulation, and sample T4 reflected the recovery phase. Immediately after collection, the samples were stored at −20°C. Cortisol concentrations were determined at the Laboratory of Endocrinology at UMC Utrecht and measured without extraction using an in‐house competitive radio‐immunoassay employing a polyclonal anticortisol‐antibody (K7348) with the tracer [1,2‐3H(N)]‐Hydrocortisone (PerkinElmer NET396250UC). The lower limit of detection was 1.0 nmol/L.

###### 2.3.4.1.3 Stress Reactivity to Manipulation

Stress reactivity (i.e., global affect and salivary cortisol reactivity) was calculated in response to the experimental manipulation with a measure of maximum increase, by selecting the lowest baseline and the highest peak. This method was chosen in accordance with previous studies assessing stress reactivity in light of individual variations (Simons et al. 2019). Specifically, global affect reactivity was calculated by subtracting global affect at baseline (VAS1) from the highest peak of global affect (VAS2 or VAS3). Salivary cortisol reactivity was calculated by subtracting the lowest baseline of salivary cortisol concentrations (sample T0 or T1) from the highest peak of salivary cortisol concentrations (sample T2 or T3).

##### 2.3.4.2 Milk Glucocorticoid Concentrations

During the lab visit, mothers were asked to collect a milk sample of approximately 6–8 mL in a tube provided by the researcher, by method of choice (i.e., using a sterilized pump or hand expression). The sample was immediately cooled in a portable freezer. At the end of the visit, when the sample was not frozen yet, a sterile pipette was used to divide each milk sample into 1.5 mL aliquots. Upon completion of the data collection period, one 1.5 mL aliquot from each participant was brought to UMC Utrecht by temperature‐controlled transport. Here, the milk samples were analyzed for cortisol and cortisone concentrations (LC–MS/MS). First, milk was centrifuged for 15 min at 14,000 RCF at 4°C. The fatty layer was removed. The liquid layer was transferred into a second tube and centrifuged for 5 min at 3000 RCF at room temperature. 0.5 mL from the liquid layer was mixed with internal standards Cortisol‐9,11,12,12‐D4 and Cortison‐D8. After 30 min of incubation, cortisol, cortisone, and its internal standards were extracted with MTBE and quantified by LC–MS/MS. The CC‐ratio (i.e., cortisol‐to‐cortisone ratio) was calculated by dividing cortisol by cortisone, with a higher ratio meaning more cortisol compared to cortisone.

##### 2.3.4.3 Mental Health Symptoms

###### 2.3.4.3.1 General Mental Health Symptoms

General mental health symptoms were measured using the following self‐report questionnaires: the Edinburgh Postnatal Depression Scale (EPDS; Cox et al. 1987), the State subscale of the State Trait Anxiety Inventory (STAI; Spielberger 1983) and the Perceived Stress Scale‐10 (PSS‐10; Cohen et al. 1983). The EPDS is a widely used questionnaire to measure depressive symptoms in the perinatal period. The questionnaire consists of 10 items covering symptoms over the past seven days, with a 4‐point scale ranging from 0 to 3. The sum score ranges from 0 to 30, with higher scores indicating higher levels of depressive feelings. The STAI State consists of 20 items on a 4‐point scale ranging from 1 to 4 and is used to measure anxiety at the moment of answering the questions. The sum score ranges from 20 to 80, with higher scores indicating higher reported anxiety levels. The PSS‐10 is a questionnaire to measure the perceived stress levels and covers the month prior to assessment. The questionnaire consists of 10 items answered on a 5‐point scale, ranging from 0 to 4, with higher scores indicating higher levels of reported stress. The sum score ranges from 0 to 40. The EPDS and STAI were assessed at two and six weeks postpartum, while the PSS‐10 was assessed only at 6 weeks. Reliability of all scales was good to excellent (McDonald's ω ranged from 0.88 to 0.94). A composite score was created based on the means of all standardized sum scores (i.e., EPDS, STAI and PSS‐10; Beijers et al. 2020). The reason for creating a composite variable is because of high intercorrelations, which can cause problems of multicollinearity when included as independent variables in the analyses. Moreover, running separate analyses per variable is accompanied by an increased risk of chance findings. Hence, a composite score can be considered the most adequate approach (Beijers et al. 2020). This composite score represented the general mental health symptoms score and was used for the analyses.

###### 2.3.4.3.2 Postpartum Specific Mental Health Symptoms

Postpartum specific mental health symptoms were assessed with the Postpartum Specific Anxiety Scale (PSAS; Fallon et al. 2016). At two weeks postpartum, participants completed a short version (PSAS‐RSF‐C; Silverio et al. 2021), while at six weeks postpartum, the full PSAS was completed. The PSAS consists of 51 items on a 4‐point scale ranging from 1 to 4 and addresses specific worries in the postpartum period. Sum scores range from 51 to 204, with higher scores indicating higher reported postpartum anxiety. The PSAS‐RSF‐C includes 12 items of the original PSAS. Sum scores range from 12 to 48. Reliability of scales was good to excellent (McDonald's ω ranged from 0.83–0.95). Postpartum specific mental health symptoms are based on the composite score consisting of the mean of both standardized sum scores (PSAS and PSAS‐RSF‐C).

##### 2.3.4.4 Confounders

Infant age in weeks and the start time of the lab visit were used as confounders.

#### 2.3.5 Missing Data

The following missingness was observed: VAS3 (*n* = 1), VAS4 (*n* = 2), T0 (*n* = 1), T1 (*n* = 3), T2 (*n* = 4), T3 (*n* = 7), milk cortisol (*n* = 2) and milk cortisone (*n* = 2). The following steps were taken to clean the data. First, the data was thoroughly screened for deviations from the study protocol. The preregistered rules to determine which data were considered valid were as follows: (1) the manipulation (i.e., TSST or control task) should be performed correctly, (2) saliva samples should be collected ±10 min from the planned time, (3) the milk sample should be collected ±10 min of the planned time, (4) VAS1, VAS2, and VAS3 should be assessed at protocol time; VAS4 needed to be assessed minimally 20 min after the end of the manipulation. Invalid data after applying these rules was recoded as missing data, namely: VAS3 (*n* = 1), VAS4 (*n* = 1), T1 (*n* = 2), T3 (*n* = 6), T4 (*n* = 5), milk cortisol (*n* = 1) and milk cortisone (*n* = 1). Second, the data was inspected for implausible salivary or milk cortisol/cortisone values. These were not found in the data. Third, the remaining data was inspected for outliers (i.e., values of more than 3 standard deviations above/below the mean). All outliers were considered valid datapoints (postpartum specific mental health symptoms *n* = 1, milk cortisol *n* = 1, milk cortisone *n* = 1). The missing data in the study variables were imputed using multiple imputation technique, as explained in section 2.3.

#### 2.3.6 Statistical Analyses

All analyses were performed in R. Following preregistration, preliminary analyses were performed to test the effectiveness of the experimental manipulation. Group comparisons between the stressor and control groups on global affect at VAS2 and VAS3 and salivary cortisol at T2 and T3 were tested, using independent samples *t*‐tests if variables were normally distributed, and Mann–Whitney *U* tests if they were not. Also, correlations between study variables were inspected.

Subsequently, the hypotheses were tested using linear regression analyses. To test the effect of the stressor and moderation of general mental health symptoms, the following variables were entered simultaneously in the regression analysis: group (stressor group = 1/control group = 0), general mental health symptoms, and the interaction terms of group × general mental health symptoms. This model was run twice, for each of the outcome variables: milk cortisol and milk cortisone. Secondly, to test stress reactivity during the experimental study (irrespective of group), the following variables were entered simultaneously in the regression analyses: stress reactivity (global affect and salivary cortisol), general mental health symptoms, infant age in weeks, start time of lab visit, and the interaction terms of stress reactivity (i.e., global affect and salivary cortisol) × general mental health symptoms. These models were repeated for each of the outcome variables: milk cortisol and milk cortisone. Notably, there was no detection of multicollinearity between global affect and salivary cortisol reactivity variables, as they were weakly correlated and the variance inflation factor was ≤ 1.57. Therefore, to conduct a more parsimonious analysis, both factors were included within a single model rather than being tested separately as initially preregistered. Additionally, we ran exploratory analyses. First, we ran the main analyses again with postpartum‐specific instead of general mental health symptoms. Second, we repeated the main analyses using CC‐ratio as an outcome. All models were inspected for homogeneity and distribution of the residuals. Outliers of model residuals were visually inspected, and their influence was tested using Cook's Distance.

## 3 Results

### 3.1 Naturalistic Study

#### 3.1.1 Preliminary Analyses

The descriptive statistics and correlations between study variables are presented in Tables 1 and 2. Global affect levels in the morning, afternoon, and evening all correlated positively with each other (all *r*'s > 0.41). Higher milk cortisol was associated with higher milk cortisone at each assessment moment (all *r*'s > 0.53). In contrast, milk cortisol measured in the morning, afternoon, and evening intercorrelated only weakly or not at all. The same was true for milk cortisone and the CC‐ratio. Global affect was not correlated with milk cortisol, cortisone, or CC‐ratio at the same moment, except for a weak positive correlation between more global affect and increased milk cortisone in the afternoon.

**TABLE 1:** Naturalistic study: Descriptives of sample and study variables.

Variables	*N*	M (SD)/%	Range
Demographics			
Age of mother (years)	110	32.40 (3.50)	22–39
Age of infant (weeks)	110	6.51 (0.47)	5.43–8.86
Sex of infant			
Female	57	48%	
Male	53	52%	
Nationality			
Dutch	102	92.73%	
Other	8	7.27%	
Educational level			
High	97	88.18%	
Low/medium	13	11.82%	
Study variables			
Global affect morning (VAS)	96	26.59 (16.28)	0.58–77.77
Global affect afternoon (VAS)	98	22.44 (16.61)	2.20–79.17
Global affect evening (VAS)	97	26.75 (18.98)	0.23–78.13
Milk cortisol morning (nmol/L)	89	12.60 (9.82)	0.68–45.43
Milk cortisol afternoon (nmol/L)	93	3.20 (2.47)	0.19–11.32
Milk cortisol evening (nmol/L)	88	0.51 (0.49)	0.20–3.08
Milk cortisone morning (nmol/L)	89	25.83 (6.58)	10.10–39.61
Milk cortisone afternoon (nmol/L)	93	16.54 (5.58)	6.08–33.78
Milk cortisone evening (nmol/L)	88	5.45 (3.18)	1.31–23.46

*Note:* nmol/L = nanomoles per liter. Educational level refers to the highest degree within the Dutch educational system. Category ‘high’ refers to ‘HBO’ and university. Category ‘low’ refers to primary school, high school, and ‘MBO’. The descriptives of the study variables are based on the cleaned raw data (i.e., without imputation of missing values).

Abbreviation: VAS, visual analogue scale.

**TABLE 2:** Naturalistic study: Pearson correlations between study variables.

	1	2	3	4	5	6	7	8	9	10	11	12
1. Global affect morning	—											
2. Global affect afternoon	0.53**	—										
3. Global affect evening	0.41**	0.63**	—									
4. Cortisol morning	0.01	0.00	0.08	—								
5. Cortisol afternoon	0.04	0.02	0.21**	−0.25**	—							
6. Cortisol evening	−0.13**	−0.03	0.00	−0.07	0.06	—						
7. Cortisone morning	0.10	0.10	0.09	0.53**	−0.35**	−0.10	—					
8. Cortisone afternoon	0.06	0.14**	0.20**	−0.20**	0.71**	−0.14**	0.08	—				
9. Cortisone evening	−0.04	0.04	0.04	0.01	−0.08	0.78**	0.17**	−0.02	—			
10. CC‐ratio morning	−0.02	−0.02	0.07	0.95**	−0.19**	−0.05	0.30**	−0.26**	−0.05	—		
11. CC‐ratio afternoon	−0.01	−0.05	0.14**	−0.21**	0.88**	0.16**	−0.52**	0.37**	−0.11**	−0.09	—	
12. CC‐ratio evening	−0.18**	−0.08	−0.03	−0.15**	0.22**	0.62**	−0.43**	−0.17**	0.07	−0.04	0.45**	—

*Note:* CC‐ratio: cortisol‐to‐cortisone ratio. These correlations are pooled correlations based on the imputed dataset.

** *p* < 0.01.

Assumptions of the regression analyses were met and there were no influential cases detected (Cook's Distance's < 1). Sensitivity analyses with complete cases rendered similar results to the analyses based on the five imputed datasets. Therefore, in this paper we will present the results based on the imputed datasets (i.e., pooled over the five imputed data sets).

#### 3.1.2 Main Analyses: Associations Between Global Affect and MGCs

The results from the main regression analyses showed no association between self‐reported global affect and MGCs (i.e., cortisol and cortisone) in the morning, afternoon, and evening (all *p*‐values ≥ 0.19; Table 3).

**TABLE 3:** Naturalistic study: Association between global affect and MGCs.

Effect	Estimate (95% CI)	Standard error	*p*
Morning cortisol (log)			
Intercept	0.97 (0.91–1.04)	0.03	0.00*
Global affect	0.03 (−0.04 to 0.10)	0.03	0.34
Time of sampling	−0.17 (−0.24 to −0.10)	0.03	0.00*
Infant age	−0.04 (−0.11 to 0.02)	0.03	0.19
*R* ^2^	0.24		
Afternoon cortisol (log)			
Intercept	0.38 (0.31–0.45)	0.03	0.00*
Global affect	0.00 (−0.07 to 0.07)	0.04	0.91
Time of sampling	0.03 (−0.05 to 0.11)	0.04	0.45
Infant age	−0.02 (−0.08 to 0.05)	0.03	0.62
*R* ^2^	0.01		
Evening cortisol			
Intercept	−0.42 (−0.48 to −0.36)	0.03	0.00*
Global affect	0.00 (−0.06 to 0.06)	0.03	0.99
Time of sampling	−0.14 (−0.20 to −0.08)	0.03	0.00*
Infant age	−0.02 (−0.08 to 0.04)	0.03	0.50
*R* ^2^	0.20		
Morning cortisone			
Intercept	26.16 (24.92–27.40)	0.62	0.00*
Global affect	0.86 (−0.48 to 2.19)	0.66	0.20
Time of sampling	−2.31 (−3.50 to −1.12)	0.60	0.00*
Infant age	−1.12 (−2.32 to 0.07)	0.60	0.07
*R* ^2^	0.16		
Afternoon cortisone			
Intercept	16.47 (15.34–17.59)	0.56	0.00*
Global affect	0.83 (−0.43 to 2.08)	0.62	0.19
Time of sampling	−0.21 (−2.16 to 1.73)	0.85	0.81
Infant age	−0.48 (−1.59 to 0.62)	0.56	0.39
*R* ^2^	0.04		
Evening cortisone			
Intercept	5.43 (4.86–6.00)	0.29	0.00*
Global affect	0.19 (−0.46 to 0.84)	0.32	0.55
Time of sampling	−1.44 (−2.18 to −0.70)	0.35	0.00*
Infant age	−0.37 (−0.95 to 0.21)	0.29	0.20
*R* ^2^	0.22		

*Note:* These are the pooled results from the linear regression analyses based on imputed datasets.

Abbreviation: MGCs, milk glucocorticoids.

* *p* < 0.05.

#### 3.1.3 Exploratory Analyses: Association Between Global Affect and Cc‐Ratio

The results from the exploratory regression analyses showed no association between global affect stress and the CC‐ratio in the morning, afternoon, and evening (all *p*‐values ≥ 0.53; Table S1).

### 3.2 Experimental Study

#### 3.2.1 Preliminary Analyses

**FIGURE 3:**
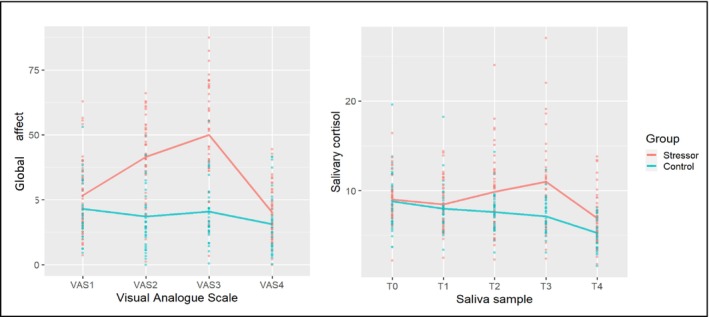
Maternal stress during the lab visit. VAS2 and VAS3 assessed global affect during the experimental manipulation. Saliva samples T2 and T3 are taken 25 min after VAS2 and VAS3, respectively, and reflect the cortisol concentrations in response to the experimental manipulation.

The descriptive statistics and the stress response during the laboratory visit are presented in Figure 3 and Table 4, and indicate that participants in the stressor group showed a significantly higher level of global affect (i.e., VAS2 and VAS3; both *p*‐values < 0.01) and salivary cortisol (i.e., T2 and T3; respectively *p* = 0.01 and *p* < 0.01) during the Trier Social Stress Test compared to the control group.

**TABLE 4:** Experimental study: Descriptives of sample and study variables.

Variables	Total			Stressor group			Control group		
	*N*	M (SD)	Range	*N*	M (SD)	Range	*N*	M (SD)	Range
Demographics									
Age of mother (years)	80	32.48 (3.44)	22–38	43	32.77 (3.88)	22–38	37	32.13 (2.86)	25–37
Age of infant (weeks)	80	8.20 (1.26)	6.29–11.57	43	8.20 (1.25)	6.29–11	37	8.20 (1.28)	6.29–11.57
Sex of infant									
Female	40	50%		20	46.5%		20	54.1%	
Male	40	50%		23	53.5%		17	45.9%	
Nationality									
Dutch	72	90%		38	88.4%		34	91.9%	
Other	8	10%		5	11.6%		3	8.1%	
Educational level in %									
High	71	88.75%		38	88.37%		33	89.18%	
Low	9	11.25%		5	11.63%		4	10.82%	
Moderators									
Anxiety complaints	80	30.64 (6.66)	20.5–52	43	31.70 (7.55)	20.5–52	37	29.41 (5.30)	20.5–42
Depressive complaints	80	5.13 (3.76)	0–16.5	43	5.92 (4.27)	0–16.5	37	4.22 (2.86)	0–11.5
Stress complaints	80	11.09 (6.11)	0–28	43	12.14 (6.61)	0–28	37	9.86 (5.30)	2–24
General MHP	80	0.00 (0.91)	−1.43 to 2.37	43	0.18 (1.03)	−1.43 to 2.37	37	−0.21 (0.71)	−1.38 − 1.53
Postpartum specific MHP	80	0.00 (0.94)	−1.53 to 2.91	43	0.17 (1.06)	−1.39 to 2.91	37	−0.19 (0.74)	−1.53 to 1.25
Global affect									
VAS1	80	24.25 (13.00)	3.63–62.82	43	26.70 (14.33)	3.63–62.82	37	21.51 (10.78)	4.53–53.00
VAS2	80	30.91 (18.32)	0.00–66.03	43	41.51 (15.70)	1.07–66.03	37	18.60 (12.60)	0.00–49.68
VAS3	78	35.95 (22.26)	0.43–87.40	41	49.93 (20.14)	3.41–87.40	37	20.44 (11.77)	0.43–55.34
VAS4	77	17.96 (11.14)	0.00–44.44	41	20.06 (10.61)	0.00–44.44	36	15.57 (11.39)	0.21–41.56
Salivary cortisol									
T0 (nmol/L)	79	8.92 (2.84)	2.2–19.6	42	9.02 (2.5)	2.2–16.4	27	8.81 (3.20)	3.7–19.6
T1 (nmol/L)	75	8.25 (2.76)	2.5–18.2	39	8.48 (2.93)	2.5–14.4	36	7.8 (2.57)	3.4–18.2
T2 (nmol/L)	76	8.81 (3.76)	2.3–24	41	9.85 (4.51)	2.3–24	35	7.6 (2.11)	3.1–14.3
T3 (nmol/L)	67	9.03 (4.48)	2.4–27	33	11 (5.45)	2.4–27	34	7.12 (1.9)	3.1–12.3
T4 (nmol/L)	75	6.15 (2.56)	1.6–13.8	40	6.92 (2.93)	1.8–13.8	35	5.28 (1.71)	1.6–8.1
Stress reactivity									
Global affect reactivity	80	22.63 (23.63)	−11.25 to 86.5	80	37.06 (22.03)	−10.75 to 86.5	80	5.85 (11)	−11.25 to 34.25
Saliva cortisol reactivity	79	1.37 (3.44)	−3.9 to 14.1	42	2.5 (4.12)	−3.5 to 14.1	37	0.01 (1.6)	−3.9 to 5.2
Outcomes									
Milk cortisol (nmol/L)	77	4.21 (5.9)	0.2–38.4	42	5.9 (7.49)	0.2–38.4	35	2.19 (1.53)	0.2–7.6
Milk cortisone (nmol/L)	77	14.74 (6.17)	3.2–28.7	42	16.33 (7.1)	3.2–28.7	35	12.83 (4.17)	3.4–20.7

*Note:* nmol/L = nanomoles per liter. The descriptives of the study variables are based on the cleaned data without imputation of missing values.

Abbreviations: MHP, mental health symptoms; T0–T4, saliva samples; VAS, visual analogue scale.

The correlations between study variables were inspected and showed that milk cortisol, cortisone, and the CC‐ratio were highly intercorrelated (Table 5). Participants in the stressor group had higher MGCs concentrations and a higher CC‐ratio compared to the control group. Increased salivary cortisol reactivity was highly correlated with increased MGCs concentrations and an increased CC‐ratio, while increased global affect reactivity was only weakly correlated with these outcomes.

**TABLE 5:** Experimental study: Pearson correlations between study variables.

	1	2	3	4	5	6	7	8
1. Group	—							
2. Global affect reactivity	0.67**	—						
3. Salivary cortisol reactivity	0.44**	0.31**	—					
4. Milk cortisol	0.31**	0.18**	0.83**	—				
5. Milk cortisone	0.27**	0.15**	0.71**	0.70**	—			
6. CC‐ratio	0.30**	0.22**	0.78**	0.97**	0.63**	—		
7. General MHP	0.21**	0.13**	−0.01	−0.10*	−0.02	−0.08	—	
8. Postpartum specific MHP	0.20**	0.25**	−0.01	−0.11*	−0.02	−0.08	0.78**	—

*Note:* Group is coded as: stressor group = 1, control group = 0. CC‐ratio: cortisol‐to‐cortisone ratio. These correlations are pooled correlations based on the imputed datasets. MHP = Mental health symptoms.

** *p* < 0.01.

* *p* < 0.05.

The assumptions of the regression analyses were met and there were no influential cases detected (Cook's Distance's < 1). Lastly, we ran two sensitivity analyses: one to compare the main analyses between complete case analyses and analyses based on the imputed datasets and, as maternal cortisol decreases following nursing (Beery et al. 2023), another to test the influence of three participants that had fed their infant prior to milk sampling. Complete case analyses (with and without these three participants) and pooled estimates of the five imputed datasets rendered similar results. Therefore, in this paper we will present the pooled results based on the imputed datasets.

#### 3.2.2 Main Analyses

##### 3.2.2.1 Effect of Acute Laboratory Stressor on Milk Glucocorticoids

**FIGURE 4:**
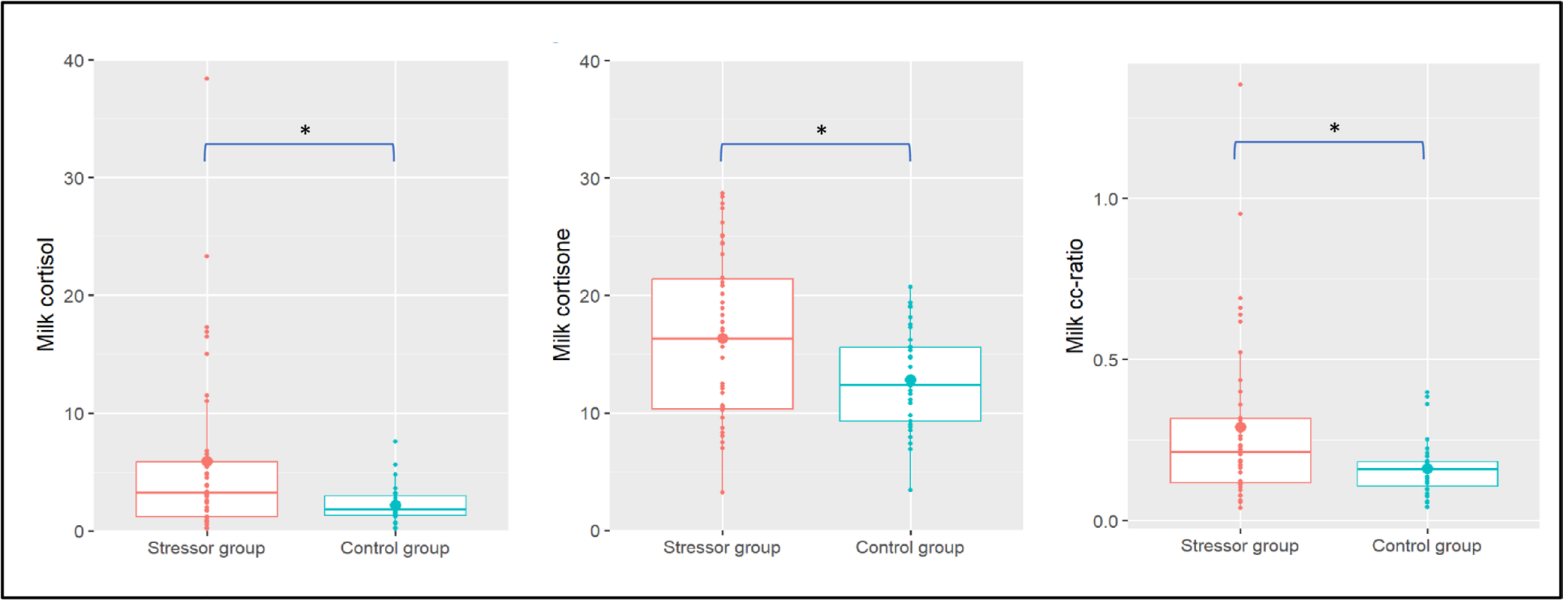
Milk glucocorticoid concentrations during the laboratory visit. CC‐ratio = cortisol‐to‐cortisone ratio. The boxplots represent the mean and interquartile ranges per group. Lines represent the difference between the stressor and control group in milk cortisol and milk cortisone (in nanomoles per liter) and the milk CC‐ratio. *p < 0.05.

Results of the regression analyses demonstrated that participants in the stressor group showed significantly higher milk cortisol and cortisone compared to the control group (both *p‐*values = 0.02; see Table 6 and Figure 4). Mental health symptoms did not moderate the effects of group on MGCs.

**TABLE 6:** Experimental study: Effect of laboratory stressor on MGCs and moderation by mental health symptoms.

	Estimate (95% CI)	Standard error	*p*
Milk cortisol (log)			
Intercept	0.58 (0.23–0.94)	0.17	0.00*
Group	0.57 (0.10–1.04)	0.24	0.02*
MHP	0.18 (0.25–0.62)	0.22	0.41
Group × General MHP	−0.35 (−0.87–0.17)	0.26	0.18
*R* ^*2*^	0.10		
Milk cortisone			
Intercept	13.11 (11.10–15.13)	1.01	0.00*
Group	3.29 (0.58–6.00)	1.36	0.02*
MHP	1.16 (−1.33–3.65)	1.25	0.36
Group × General MHP	−2.33 (−5.29–0.63)	1.48	0.12
*R* ^2^	0.11		

*Note:* Results from linear regression analyses. Group is coded as stressor group = 1, control group = 0.

Abbreviations: MGCs, milk glucocorticoids; MHP, mental health symptoms.

* *p* < 0.05.

##### 3.2.2.2 Association Between Stress Reactivity and MGCs

**FIGURE 5:**
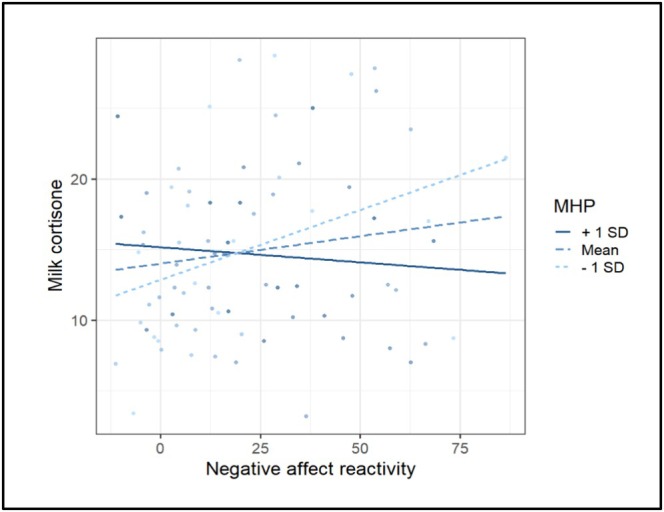
Moderation of mental health symptoms. MHP, mental health symptoms.

Regression analyses revealed that higher salivary cortisol reactivity (irrespective of group) was strongly associated with increased MGCs (i.e., milk cortisol and cortisone), while no moderation of mental health symptoms was found (Table 7). Instead, global affect reactivity (irrespective of group) was not significantly associated with milk cortisol or cortisone, although we did find a significant interaction effect with mental health symptoms (Figure 5). Namely, the positive association between global affect reactivity and milk cortisone seemed to be only present for mothers with relatively fewer mental health symptoms. Indeed, the slope of the association between global affect reactivity and milk cortisone for the women with the highest quartile of mental health symptoms was not significant (*p* = 0.46), while the slope of the women with the lowest quartile of mental health symptoms was marginally significant (*p* = 0.06).

**TABLE 7:** Experimental study: Association between stress reactivity and MGCs and moderation by mental health symptoms.

Effect	Estimate (95% CI)	Standard error	*p*
Cortisol (log)			
Intercept	2.83 (0.60–5.07)	1.12	0.01
Global affect reactivity	0.00 (−0.20 to 0.20)	0.10	0.99
Salivary cortisol reactivity	0.77 (0.56–0.98)	0.10	0.00**
General MHP	0.00 (−0.18 to 0.18)	0.09	0.97
Time of sampling	−5.00 (−8.44 to −1.55)	1.72	0.01*
Infant age	0.09 (−0.06 to 0.24)	0.08	0.23
Global affect reactivity × General MHP	−0.04 (−0.22 to 0.15)	0.09	0.68
Salivary cortisol reactivity × General MHP	0.09 (−0.16 to 0.33)	0.12	0.47
*R* ^2^	0.54		
Cortisone			
Intercept	27.42 (15.54–39.30)	5.95	0.00
Global affect reactivity	−0.65 (−1.72 to 0.43)	0.54	0.23
Salivary cortisol reactivity	4.54 (3.38–5.70)	0.58	0.00*
General MHP	−0.01 (−0.98 to 0.95)	−0.03	0.98
Time of sampling	−24.49 (−43.37 to −5.61)	9.45	0.01*
Infant age	0.09 (−0.71 to 0.88)	0.40	0.83
Global affect reactivity × General MHP	−1.13 (−2.14 to −0.11)	0.51	0.03*
Salivary cortisol reactivity × General MHP	0.55 (−0.75 to 1.85)	0.65	0.40
*R* ^2^	0.58		

*Note:* Results from linear regression analyses. Global affect reactivity is based on self‐reported global affect using visual analogue scales, while salivary cortisol reactivity is based on salivary cortisol concentrations.

Abbreviations: MGCs, milk glucocorticoids; MHP, mental health symptoms.

* *p* < 0.05.

** *p* < 0.01.

#### 3.2.3 Exploratory Analyses

Exploratory analyses demonstrated that participants in the stressor group had a significantly higher CC‐ratio compared to the control group (Figure 4 and Table S2). Also, heightened salivary cortisol reactivity was significantly related to an increased CC‐ratio, while global affect reactivity was not related to the CC‐ratio. Repetition of the main and exploratory analyses with postpartum‐specific mental health symptoms instead of general mental health symptoms as a moderator revealed no significant interaction effects (all *p*‐values ≥ 0.21, Table S3).

## 4 Discussion

The goal of this study was to determine the impact of maternal stress on MGCs (i.e., milk cortisol and cortisone) and the CC‐ratio (i.e., cortisol‐to‐cortisone ratio) by means of a naturalistic and experimental study. In the naturalistic study, self‐reported current global affect was not related to concentrations of MGCs nor the CC‐ratio in the same moment (i.e., morning, afternoon, evening). In the experimental study (i.e., RCT), the stressor group showed significantly higher concentrations of post‐stressor MGCs and a higher CC‐ratio compared to the control group. Similarly, heightened salivary cortisol reactivity during the laboratory visit, irrespective of group, was significantly associated with increased post‐stressor MGCs concentrations as well as an increased CC‐ratio. Heightened global affect reactivity did not appear to be associated with concentrations of post‐stressor MGCs or the CC‐ratio, although it was associated with increased milk cortisone in mothers with low levels of mental health symptoms.

To our knowledge, this is the first study showing that exposure to an acute psychosocial stressor (i.e., TSST) leads to an increase in post‐stressor MGCs in humans. These findings are in line with animal studies, which found an increase in milk glucocorticoids after (artificial) stress induction (Termeulen et al. 1981; Yeh 1984). Similarly, we found that heightened salivary cortisol reactivity (irrespective of group) during the laboratory visit was associated with increased post‐stressor MG, which is consistent with previous research indicating that milk cortisol originates from maternal circulation (Beery et al. 2023; van der Voorn et al. 2016). The current study adds to the field by finding evidence for a causal link between stressor exposure and MGCs, including the CC‐ratio. Replication studies are warranted.

As MGCs are ingested by infants, an important question for future research is whether MGCs exert long‐term programming effects on child development, and if this were the case, what the nature of these effects is. It is also of interest to determine if and to what extent maternal exposure to (chronic) stressor(s) affects these potential programming effects. These effects could occur through absorption in the intestines, influencing intestinal maturation, or by crossing the intestinal barrier into the bloodstream, where MGCs may exert systemic effects (Ellsworth et al. 2018; Hollanders et al. 2017). Indeed, prior research with animals as well as humans found first evidence for a link between MGCs and offspring outcomes (de Weerth et al. 2022). Also, higher cortisol concentrations in milk have been associated with temperamental differences in infants (Grey et al. 2013; Nolvi et al. 2018). However, other studies do not find evidence for programming effects by milk cortisol (Hechler et al. 2018; Toorop et al. 2020) and more importantly, potential effects of milk cortisone are even less understood as there is a lack of studies examining this. It remains unknown if ingested milk cortisone is absorbed by the gut in a similar way as milk cortisol, and if it can be converted back to cortisol within the infant's body. Hence, our understanding of the potential long‐term developmental significance of variations in MGCs and the CC‐ratio remains speculative and future research is needed. Moreover, the potential impact on child development of not being exposed to MGCs (i.e., when being fed with formula instead of breast milk) also remains unknown.

In addition, we found little evidence for an association between psychological stress and MGCs. More specifically, within the naturalistic study, there was no association between current global affect and MGCs. Since mothers from this low‐risk sample reported on average low levels of global affect, it is possible that natural fluctuations in milder stress might not lead to detectable differences in MGCs. This is supported by prior studies (Lindberg et al. 2021; Zielinska‐Pukos et al. 2022), including the recent work by Vacaru et al. (2023), who showed that maternal mental health symptoms in a healthy community sample were not predictive of milk cortisol concentrations in samples taken at home (Vacaru et al. 2023). Low global affect levels may also result from the relaxing effects of breastfeeding. That is, breastfeeding has been linked with psychological and physiological adaptations in mothers, such as reduced anxiety and cortisol levels, which are associated with circulating oxytocin levels (Uvnäs Moberg et al. 2020).

However, the reasoning that milder stress might not lead to detectable differences in MGCs does not apply to our findings within the experimental study, in which the mothers showed the expected psychological response to the TSST and reported significantly higher levels of global affect compared to the naturalistic study. However, again we did not observe a main effect of self‐reported global affect reactivity on post‐stressor MGCs or the CC‐ratio. Since absence of evidence is not the same as evidence of absence, future research is needed with larger samples to clarify if there is a link between psychological stress (reactivity) and MGCs, or not.

Importantly, the relationship between psychological stress and MGCs may only be evident in a specific subgroup—those who are not facing mental health symptoms. Namely, in our experimental study, we found that heightened global affect reactivity was associated with increased milk cortisone only among mothers with low levels of mental health symptoms. This finding does not support our hypothesis that there would be higher HPA axis reactivity in mothers with high levels of mental health symptoms. However, as we did not find a similar moderation by mental health symptoms in the analyses with milk cortisol or the CC‐ratio, dysregulation of the HPA axis is unlikely to explain these findings. Another possible explanation for the moderation by mental health symptoms may be that there were differences in milk sampling times between mothers with high and low mental health symptoms, as the laboratory procedure could have been influenced by their mental state, including relaxation, focus, or anxiety. Variations in sampling times could potentially affect MGCs measurements, given that the cortisol to cortisone conversion by the enzyme 11β‐HSD2 likely peaks about 15 min post‐stressor, as seen in saliva (Bae et al. 2019). However, this explanation appears unlikely, as post hoc analyses revealed no association between milk sampling time and the level of mental health symptoms. It is also possible that prolonged exposure to mental health symptoms might influence milk cortisol and cortisone concentrations through different mechanisms, as mental health symptoms also seem to reduce the relaxing effects following the oxytocin release during breastfeeding (Stuebe et al. 2013). Further research is needed to replicate our findings and clarify how MGCs and the CC‐ratio are affected by maternal global affect reactivity and mental health issues. A within‐person study design including baseline sampling of MGCs could shed light on these questions, and this was the original intention for Study 2. However, adding an additional milk sample to the protocol was considered unfeasible given that both the vulnerability of the target population (mothers with very young baby's) and the Covid‐19 pandemic called for the visit to the lab to be as short as possible. Also, a tool that assesses the full range of mental health symptoms in low‐risk samples in the postpartum period could contribute to a more valid reflection of mental health status and would lower participant burden due to multiple questionnaires.

This study has multiple strengths. It is the first study to investigate moment‐to‐moment links between maternal stress and MGCs concentrations. In addition, the study included various types of maternal stress. The innovative study designs allowed us to collect ecologically valid home data and enabled us to test causality in the laboratory. These studies required an instrument that could capture fluctuations in mood within shorter periods of time, which led to using another scale as compared to previous studies examining the association between maternal stress and MGCs (e.g., EPDS, PSS‐10). This limits the comparability between these studies. Moreover, despite previous studies using the Global Affect scale (Monk 1989) to assess psychological stress (e.g., García Pagès et al. 2023), and visual analogue scales (VAS) being recommended to assess short‐term fluctuations in stress (Narvaez Linares et al. 2020), the use of more specific stress‐related terms might have rendered stronger relationships with MGCs. Future studies may wish to explore the combination of the Global Affect scales with additional terms such as ‘stress’, ‘nervousness’, ‘anxiety’ that may represent the concept of stress better and lead to closer associations with MGCs. Also, a limitation of this study is that the sample primarily consisted of highly educated, white, and Western mothers. Specifically, this study is based on Dutch mothers, entitled to 10–12 weeks postpartum paid maternity leave. Hence, all measurements within the naturalistic and experimental study took place during the maternity leave of the participants. This limits the generalizability to countries with different lengths of (paid) maternity leave. Given that the work resumption period after maternity leave is associated with various challenges and resulting stress for many mothers (Beijers 2024; Okorn et al. 2024), this raises the question of whether mothers feel and respond differently to daily stressors once they return to work. Another limitation of the study is that our studies were designed to detect medium effect sizes, making them potentially underpowered to detect smaller effect sizes. Moreover, the power of some analyses to detect such medium effect sizes was compromised. While we combined global affect and salivary cortisol reactivity within one model to be more parsimonious, this led to the inclusion of seven predictors in the analyses investigating stress reactivity irrespective of group and subsequently decreased power for finding medium effect sizes (power of 0.66). The data collection occurred during the COVID‐19 pandemic which might have affected our results. Lastly, to minimize burden in the naturalistic study, participants were allowed to choose their own data collection day. Future research should consider randomizing the collection day to better capture a representative day of these mothers' daily lives.

## 5 Conclusion

To conclude, this study showed that exposure to an acute laboratory stressor caused an increase in milk cortisol, cortisone, and the cortisol‐to‐cortisone ratio. Similarly, higher MGCs concentrations and a higher CC‐ratio were observed in cases of higher salivary cortisol reactivity. However, we found little evidence that global affect (reactivity) was associated with MGCs and the CC‐ratio. Only when taking into account mental health symptoms during the past week(s), milk cortisone was associated with global affect reactivity. This result warrants further investigation, including on HPA axis functioning to elucidate the interaction between acute and chronic stress and its impact on MGCs. Larger studies are essential to investigate the factors influencing milk glucocorticoid (MGC) concentrations, their interactions with maternal acute and daily stress, and their effects on offspring outcomes such as intestinal maturation and behavioral development. Understanding how maternal, infant, and environmental factors shape MGC levels is particularly important given that formula‐fed infants are not exposed to these bioactive compounds. Such research is critical for uncovering potentially adaptive and non‐adaptive short‐ and long‐term effects of MGCs on child health and development.

## Author Contributions

**H. Lustermans:** conceptualization, data curation, formal analysis, investigation, methodology, project administration, visualization, writing – original draft. **R. Beijers:** conceptualization, funding acquisition, methodology, supervision, writing – review and editing. **C. de Weerth:** conceptualization, funding acquisition, methodology, supervision, writing – review and editing.

## Ethics Statement

This study was approved by the Ethics Committee of the Faculty of Social Sciences of the Radboud University under the blanket research line ‘pregnancy‐4years Developmental Psychobiology Lab’ (SW2017‐1303‐497), including two amendments (ECSW‐2019‐051 and ECSW‐2020‐021).

## Conflicts of Interest

The authors declare no conflicts of interest.

## Supporting information


**Table S1:** psyp70150‐sup‐0001‐supinfo.docx.

## Data Availability

Data described in the manuscript will be made available upon request pending application and approval.
