# Supplementary material for: Investigating the Impact of Maternal Stress on Milk Glucocorticoids: A Multimethod Approach
Source: Psychophysiology. 2025 Sep 16;62(9):e70150. doi: 10.1111/psyp.70150 (PMC12439325; doi:10.1111/psyp.70150)
Supplement: Supplementary file 1 — Table S1: psyp70150‐sup‐0001‐supinfo.docx. [file PSYP-62-e70150-s001.docx]

**Supplementary files**

**Supplementary Table S1***Naturalistic study – association global affect and cc-ratio*

| **Effect** | Estimate (95% CI) | Standard Error | *p-V*alue |
| --- | --- | --- | --- |
| **Morning cc-ratio** |  |  |  |
| Intercept | .46 (.39 – .54) | .04 | .00 |
| Global affect | .00 (-.06 – .07) | .03 | .89 |
| Time of sampling | -.11 (-.18 – -.05) | .03 | .00** |
| Infant age | -.04 (-.11 – .02) | .03 | .18 |
| R² | .14 | | |
| **Afternoon cc-ratio** |  |  |  |
| Intercept | .18 (.16 – .20) | .01 | .00 |
| Global affect | -.01 (-.03 – .01) | .01 | .53 |
| Time of sampling | .01 (-.01 – .03) | .01 | .41 |
| Infant age | .00 (-.02 – .02) | .01 | .85 |
| R² | .01 | | |
| **Evening cc-ratio** |  |  |  |
| Intercept | .09 (.08 – .10) | .01 | .00 |
| Global affect | .00 (-.01 – .01) | .01 | .79 |
| Time of sampling | -.01 (-.02 – .00) | .01 | .20 |
| Infant age | .00 (-.01 – .01) | .01 | .99 |
| R² | .02 | | |

*Note*. ** *p* < .01. Pooled results from exploratory linear regression analyses based on the imputed datasets.

**Supplementary Table S2***Experimental study: effect acute stressor and stress reactivity on cc-ratio and moderation by mental health symptoms*

| **Effect** | Estimate (95% CI) | Standard Error | *p-V*alue | |
| --- | --- | --- | --- | --- |
| **Laboratory stressor** | | | | |
| **CC-ratio** | | | | |
| Intercept | .16 (.10 – .23) | .03 | .00 | |
| Condition | .13 (.04 – .22) | .05 | .01* | |
| MHP | .00 (-.08 – .09) | .04 | .93 | |
| Condition*MHP | -.05 (-.15 –.05) | .05 | .33 | |
| R² | .12 | | | |
| **Stress reactivity** | | | | |
| **CC-ratio** | | | | |
| Intercept | .03 (-.35 – .41) | .19 | | .86 |
| Global affect reactivity | -.01 (-.04 – .03) | .02 | | .72 |
| Salivary cortisol reactivity | .18 (.14 – .21) | .02 | | .00** |
| MHP | -.01 (-.04 – .02) | .02 | | .39 |
| Time of sampling | -.03 (.62 –.56) | .30 | | .92 |
| Infant age | .03 (.00 – .05) | .01 | | .06 |
| Global affect reactivity*MHP | .02 (-.01 –.05) | .02 | | .18 |
| Salivary cortisol reactivity*MHP | .00 (-.04 –.04) | .02 | | .90 |
| R² | .64 | | | |

*Note*. **p* < .05. Pooled results from exploratory linear regression analyses based on the imputed datasets. MHP = Mental health symptoms.

**Supplementary Table S3***Experimental study: effect acute stressor and stress reactivity on MGCs and moderation by postpartum specific MHP*

| **Effect** | Estimate (95% CI) | Standard Error | *p-V*alue |
| --- | --- | --- | --- |
| **Laboratory stressor** | | | |
| **Cortisol (log)** | | | |
| Intercept | .57 (.22 – .92) | .18 | .00 |
| Condition | .57 (.09 – 1.04) | .24 | .02* |
| Postpartum MHP | .16 (-.31 – .61) | .23 | .51 |
| Condition*postpartum MHP | -.29 (-.85 – .27) | .28 | .31 |
| R² | .09 | | |
| **Cortisone (log)** | | | |
| Intercept | 12.97 (10.94 – 14.99) | 1.01 | .00 |
| Condition | 3.37 (.64 – 6.09) | 1.37 | .02* |
| Postpartum MHP | .62 (-2.05 – 3.28) | 1.34 | .65 |
| Condition*Postpartum MHP | -1.59 (-4.76 – 1.59) | 1.59 | .32 |
| R² | .09 | | |
| **Stress reactivity** | | | |
| **Cortisol (log)** | | | |
| Intercept | 2.81 (.56 – 5.03) | 1.10 | .01 |
| Global affect reactivity | -.01 (-.21 – .20) | .10 | .94 |
| Salivary cortisol reactivity | .80 (.59 – 1.01) | .10 | .00** |
| Postpartum MHP | .02 (-.19 – .23) | .10 | .86 |
| Time of sampling | -5.14 (-8.58 – -1.71) | 1.72 | .00** |
| Infant age | .10 (-.05 – .25) | .07 | .18 |
| Global affect reactivity*Postpartum MHP | .01 (-.18 – .20) | .11 | .95 |
| Salivary cortisol reactivity*Postpartum MHP | .13 (-.09 –.35) | .10 | .23 |
| R² | .55 | | |
| **Cortisone** | | | |
| Intercept | 27.33 (15.25 – 39.41) | 6.05 | .00 |
| Global affect reactivity | -.64 (-1.77 – .50) | .57 | .27 |
| Salivary cortisol reactivity | 4.72 (3.52 – 5.91) | .60 | .00** |
| Postpartum MHP | .20 (-.93 – 1.32) | .56 | .73 |
| Time of sampling | -25.49 | 9.68 | .01* |
| Infant age | .16 | .41 | .70 |
| Global affect reactivity*Postpartum MHP | -.38 | .53 | .47 |
| Salivary cortisol reactivity*Postpartum MHP | .66 | .61 | .29 |
| R² | .56 | | |

*Note*. **p* < .05, ** *p* < .01. Pooled results from exploratory linear regression analyses based on the imputed datasets. Postpartum MHP = Postpartum specific mental health symptoms.

|  | **Milk cortisol** | | | **Milk cortisone** | | |
| --- | --- | --- | --- | --- | --- | --- |
|  | Estimate | SE | *p* | Estimate | SE | *p* |
| Fixed effects |  |  |  |  |  |  |
| Intercept | 1.496 | 0.344 | 0.000 | 34.875 | 5.168 | 0.000 |
| Time | -2.184 | 0.553 | 0.000 | -36.307 | 8.309 | 0.000 |
| Global affect | 0.000 | 0.001 | 0.516 | 0.012 | 0.009 | 0.236 |
|  |  |  |  |  |  |  |
|  | Variance | SD |  | Variance | SD |  |
| Random effects |  |  |  |  |  |  |
| Intercept | 0.000 | 0.000 |  | 0.000 | 0.000 |  |
| Time |  |  |  | 0.000 | 0.000 |  |

**Supplementary Table S4**

*Naturalistic study: explorative multilevel modelling on association between global affect and milk glucocorticoids
Note*. **p* < .05, ** *p* < .01.
